# Echocardiographic Pulmonary Hypertension in Patients Positive for Myositis-Specific and Myositis-Associated Antibodies

**DOI:** 10.3390/jcm15010077

**Published:** 2025-12-22

**Authors:** Kristina Akopyan, Jessica Peterson, Oluyemisi Amoda, Majd Khasawneh, Susheela Hadigal, Christopher Harden, Diana Gomez Manjarres, Raju Reddy, Faye Pais

**Affiliations:** 1 Division of Hospital Medicine, Department of Medicine, University of Florida, Gainesville, FL 32610-0238, USA; 2 Malcom Randall Department of Veterans Affairs Medical Center, Gainesville, FL 32610-0238, USA; 3 Department of Medicine, Division of Pulmonary, Critical Care and Sleep Medicine, University of Florida, Gainesville, FL 32610-0238, USA; 4 Department of Medicine, Division of Pulmonary and Critical Care Medicine, University of Texas at Austin, Austin, TX 78712, USA

**Keywords:** pulmonary hypertension, myositis-specific antibody, myositis-associated antibody, interstitial lung disease

## Abstract

**Background:** The prevalence of pulmonary hypertension (PH) in patients who are positive for myositis-specific antibody (MSA) and myositis-associated antibody (MAA) remains unclear. **Methods:** We conducted a retrospective study of patients with an age of 18 years or older diagnosed with myositis interstitial lung disease (ILD) at our university’s ILD clinic between 2019 and 2022. Echocardiographic PH was defined by tricuspid regurgitation velocity (TRV) ≥ 2.9 m/s on transthoracic echocardiography (TTE) consistent with intermediate probability of PH using 2022 European Society of Cardiology/European Respiratory Society (ESC/ERS) guidelines. We grouped patients based on low probability of PH vs. intermediate to high probability of PH. We examined 6 min walk test (6MWT) data, pulmonary function tests (PFTs), all-cause mortality, and rate of lung transplantation. We also evaluated patients who were on immunosuppression vs. those not on immunosuppression. **Results:** The intermediate to high probability of PH group had a higher prevalence of dermato-specific antibodies (14.2% vs. 34.5%, *p* = 0.048). Specifically, MDA-5 was found to be more prevalent in patients with intermediate to high probability of PH (7.1% vs. 24.1%, *p* = 0.040). There was no difference in 6MWT parameters between groups (363.2 ± 115.6 m vs. 294.9 ± 147.5 m, *p* = 0.108). FVC and DLCO were lower in patients with intermediate to high probability of PH (71.3 ± 22.4 L vs. 58.8 ± 16.7 L, *p* = 0.037; 56.3 ± 21.8 mL/min/mmHg vs. 36.9 ± 15.5 mL/min/mmHg, *p* = 0.003). The all-cause mortality and rate of lung transplantation was higher in the intermediate to high probability of PH group (5.4% vs. 20.7%, *p* = 0.041, 0% vs. 6.9%, *p* = 0.049). There was no difference in all-cause mortality between patients who were on immunosuppression vs. those who were not on immunosuppression in patients with intermediate to high probability of PH (33.3% vs. 7.1%; *p* = 0.169). **Conclusions:** Patients with MSA/MAA may have an increased risk of PH with reduced lung function, higher mortality, and greater rate of lung transplantation. Our study further elucidates the growing body of evidence that dermato-specific antibodies, such as MDA-5 are associated with an increased risk of PH. Further research is needed to investigate the role of PH and immunosuppression in these patients.

## 1. Introduction

Interstitial lung disease (ILD) has a high prevalence in patients with myositis-specific antibody (MSA) and myositis-associated antibody (MAA). Pulmonary hypertension (PH) is a condition that leads to increased morbidity and mortality in patients with ILD. PH is associated with connective tissue diseases (CTDs), but it is not typically observed in myositis [1]. The prevalence of myositis is estimated to be only 4% in CTD-PH cohorts of patients in the United Kingdom (UK) and Korea [2]. The prevalence of PH in patients positive for MSA and MAA remains unclear. PH associated with MSAs/MAAs has been interpreted as a complication of ILD in patients with idiopathic inflammatory myositis (IIM). However, there may be a role of autoimmune disease in PH associated with MSA/MAA [3].

PH associated with CTD is the second most common cause of pulmonary arterial hypertension (PAH). Systemic scleroderma (SSc) is the most common cause of CTD-associated PH with prevalence estimated to be 7.8–12%, which is followed by systemic lupus erythematosus (SLE) at 0.5–17.5% and mixed connective tissue disease (MCTD) at 5.9–8% [4,5]. PH can also occur with rheumatoid arthritis (RA), primary Sjogren’s syndrome (pSS), inflammatory myositis, and even other rheumatic diseases such as Bechet’s disease and adult-onset Still’s disease but is less common [2]. The pathogenesis of CTD-PH can be explained by endothelin 1, nitric oxide, and prostacyclin pathways. Inflammation and autoimmunity are also thought to play a role in the progression of CTD-PH [6]. The 2022 European Society of Cardiology/European Respiratory Society (ESC/ERS) guidelines recommend obtaining annual echocardiography for patients with symptomatic SSc. However, there are no established guidelines for screening for PH in other CTDs [7]. CTD-PH treatment can be divided into vasodilator PH treatment and immunosuppressive therapy. There are no randomized clinical trials on the efficacy of immunosuppression in patients with CTD-PH. However, there have been several case series which have shown the effect of immunosuppression in patients with SLE and MCTD [8].

Given the lack of data in the role of PH and immunosuppression in patients with MSA/MAA, we aimed to describe the relationship between MSA/MAA with the development of suspected PH in patients with myositis-related ILD and to evaluate how the treatment with immunosuppression may influence the progression of PH in these patients. Our hypothesis was that patients with MSA/MAA would have a higher prevalence for risk of PH in patients with myositis-related ILD. We also predicted that patients who were treated with immunosuppression could potentially improve their 6 min walk test (6MWT) parameters and pulmonary function tests (PFTs). Preliminary results from this analysis were presented at the American Thoracic Society International Conference 2025.

## 2. Methods

Our study was approved by our university’s Institutional Review Board (202300658) on 1 February 2024. Research began in March of 2024. The study data are available upon request.

### 2.1. Study Population

We conducted a retrospective study of patients with an age of 18 years or older diagnosed with myositis ILD at our university’s ILD clinic between 2019 and 2022. All patients with the following positive antibodies were included: Jo1, PL7, Pl12, EJ, OJ, MDA5, Mi2, SRP, HMGCR, TIF1, NXP2, SAE, PM-Scl, U1RNP, Ku, SSA, SSB, Ro 52, Ro 60, Cn1A. We then screened all patients randomly for those who had a tricuspid regurgitation velocity (TRV) of 2.9 m/second (m/s) or greater on transthoracic echocardiogram (TTE) consistent with intermediate probability of PH [9]. Exclusion criteria were patients with known diagnosis of other CTDs such as SSc, SLE, pSS, or RA. Based on high-resolution computed tomography (HRCT) scan of the chest, we identified patients with and without ILD.

### 2.2. Data Collection

Variables such as age, sex, race, body mass index (BMI), smoking history, clinical diagnosis, obstructive sleep apnea (OSA), computed tomography (CT) of chest findings, serologies, PFTs, 6MWT data, echocardiography, right heart catheterization (RHC) data, treatment with immunosuppression, and clinical outcomes such as death and/or lung transplantation were collected.

### 2.3. PFTs

We included forced expiratory volume in 1 s (FEV1), forced vital capacity (FVC), diffusion capacity of the lungs for carbon monoxide (DLCO), and total lung capacity (TLC).

### 2.4. 6MWT

We included 6 min walk distance walked, oxygen used, and change in dyspnea score.

### 2.5. Echocardiography

We classified patients into low and intermediate to high probability of PH based on Doppler echocardiographic measurement of TRV using 2022 ESC/ERS guidelines [9]. TRV ≤ 2.8 m/s was defined as low probability of PH. TRV ≥ 2.9 m/s was defined as intermediate to high probability of PH. We also collected data including left atrial (LA) size, right ventricular systolic pressure (RVSP), and tricuspid annular plane systolic excursion (TAPSE).

### 2.6. RHC

We then classified patients with right heart catheterization (RHC) data into patients with and without PH. We included only patients who had both TTE and RHC data and eliminated those with a RHC but no TTE. PH was defined using 2022 ESC/ERS guidelines. Pre-capillary PH was defined by mean pulmonary arterial pressure (mPAP) > 20 mmHg, pulmonary artery occlusion pressure (PAOP) ≤ 15 mmHg, and pulmonary vascular resistance (PVR) > 2 Woods units (WU). Post-capillary PH was defined by mPAP > 20 mmHg and PAOP > 15 mmHg. We also collected data on right atrial pressure (RAP), RVSP, right ventricular diastolic pressure (RVDP, PAOP, thermo-dilution cardiac output (TDCO), mPAP, cardiac index (CI), PVR, stroke volume (SV), and RAP/PAOP.

### 2.7. Statistical Analysis

All statistical analyses were performed using IBM SPSS Statistics (Version 29.0.0). Normality of continuous variables was assessed. As data demonstrated approximately normal distribution, parametric tests were applied. Continuous variables were summarized using means and standard deviations (SDs), and comparisons between groups were evaluated using independent samples *t*-tests. Categorical variables were summarized using frequencies and percentages, and group differences were evaluated using Fisher’s Exact Tests due to small cell counts. Patients were categorized into two groups based on echocardiographic probability of PH defined by TRV. Low probability of PH was defined as ≤ 2.8 m/s, and intermediate to high probability of PH was defined as ≥2.9 m/s. A subgroup analysis was performed among individuals with intermediate-high probability of PH to examine associations with immunosuppressive therapy (on immunosuppression vs. not on immunosuppression). To evaluate whether PH probability and immunosuppression had an interactive effect on physiologic performance, a two-way between subjects ANOVA was conducted for 6MWT variables (distance, dyspnea score change, and oxygen use). All statistical tests were two-tailed, and significance was defined as *p* < 0.05.

## 3. Results

A total of 214 patients were obtained using the MSA/MAA of interest. Of those patients, 85 had a TTE with TRV documented and became our analytical sample. There were 56/85 (65.9%) of patients in the low probability of PH group, while 29/85 (34.1%) were in the intermediate to high probability of PH group. There were 57.1% of males with intermediate to high probability of PH compared to 22.8% of females. This contrasted the low probability of PH group with 22.8% males and 77.2% females (*p* = 0.002). There was a higher prevalence of smoking history at 51.7% in the intermediate to high probability of PH group compared to 21.4% in the low probability of PH group (*p* = 0.004). There was no difference in age, race, BMI, or presence of OSA between groups (*p* = 0.065, 0.383, 0.610, 0.662). There was also no difference between groups in the clinical diagnosis of patients including antisynthetase syndrome (ASSD), dermato-myositis (DM), and polymyositis (PM) (*p* = 0.443, 0.392, 0.686). ILD was more prevalent in the intermediate to high probability of PH group at 93.1% compared to 75% in the low probability of PH group (*p* = 0.043) (Table 1).

We examined the prevalence of MSA/MAA in patients who had intermediate to high probability of PH documented by TRV on TTE, with and without ILD. These patients had a higher prevalence of dermato-specific antibodies (34.5%, *p* = 0.048), which includes Jo-1, Mi-2, MDA-5, TIF-1, and NXP2. There were no differences found in the presence of MSA, MAA, or ASSD antibodies (*p* = 0.073, 0.361, 0.332). The most prevalent antibodies overall in our cohort of patients were SSA52 (27.1%) followed by Jo1 (15.3%), and MDA5 (12.9%) (Table 2).

We also evaluated LA size, RVSP, and TAPSE. There was a significant difference between TAPSE with low probability PH group having a mean TAPSE of 23 mm and intermediate to high probability PH group having mean TAPSE of 21.1 mm (*p* = 0.029). For RHC parameters, we evaluated RAP, RV systolic pressure, RV diastolic pressure, PAOP, TDCO, MPAP, CI, PVR, SV, and RAP/PAWP. There was RHC data available for 30 patients, but 10 of those did not have TTE data and were eliminated. There was no significant difference in MPAP between the low and intermediate to high probability PH group (*p* = 0.840). However, there were a total of 8/30 (26.7%) patients with confirmed group I PH. There was a significant difference with RAP/PAOP with low probability PH group having mean 0.6 and high probability PH group having mean 0.9 (*p* = 0.048) (Table 3).

**Figure 1:**
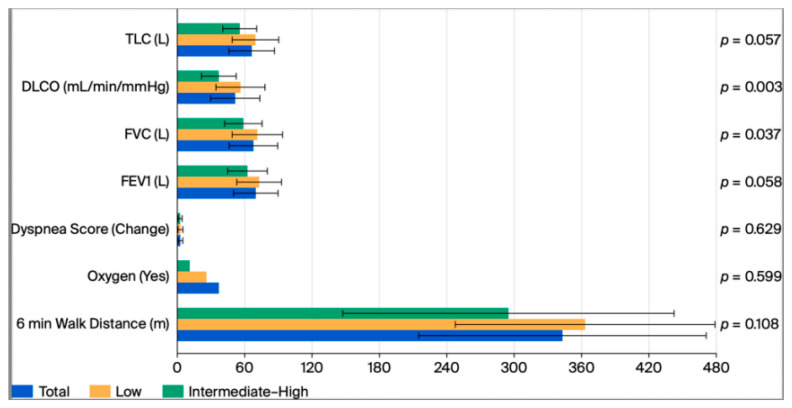
6MWT and Pulmonary Function Test of whole sample and grouped differences between low probability of PH and intermediate to high probability of PH. *T*-test was used for all variables except oxygen use where Fisher’s Exact Test was used. Statistical significance is defined as *p* value < 0.05.

**Figure 2:**
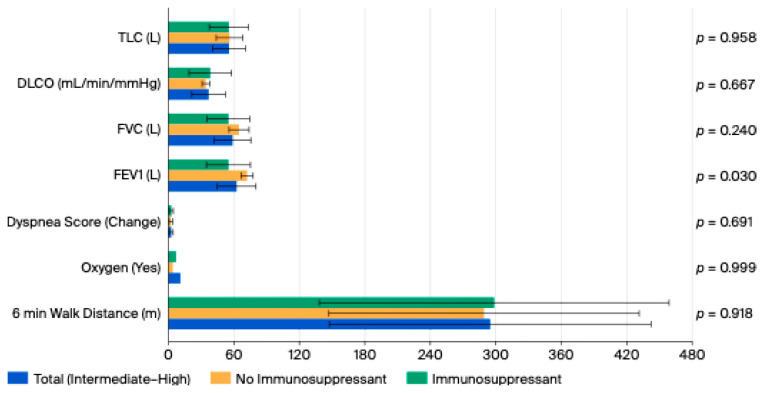
6MWT and Pulmonary Function Test of those with intermediate-high probability of PH and grouped differences between those on immunosuppression vs. those who are not. *T*-test was used for all variables except oxygen use where Fisher’s Exact Test was used. Statistical significance is defined as *p* value < 0.05.

We evaluated 6MWT distance walked, oxygen used, and change in dyspnea score between low and intermediate to high probability of PH groups and found no differences between the groups (*p* = 0.108, 0.599, 0.629). There was also no difference between 6MWT distance, oxygen used, and dyspnea score in patients on immunosuppression compared to those not on immunosuppression in the intermediate to high probability of PH group (*p* = 0.918, 0.999, 0.691). Probability of PH and immunosuppression had no overall effect on 6MWT distance walked and change in dyspnea score (*p* = 0.273, 0.873) However, there was a significant model effect of PH probability and immunosuppression on oxygen used (*p* = 0.045) (Table 4, Table 5 and Table 6 and Figure 1 and Figure 2).

We examined the differences between PFTs including percentage predicted of FEV1, FVC, DLCO, TLC, and FVC/DLCO. FVC and DLCO were lower in patients with intermediate to high probability of PH (*p* = 0.037, *p* = 0.003). There was no difference between FVC, DLCO, and TLC in patients who were on immunosuppression vs. those who were not on immunosuppression in the intermediate to high probability of PH group (*p* = 0.240, 0.667, 0.958). However, there was a difference in FEV1 with those on immunosuppression having a lower FEV1 (*p* = 0.030) (Table 4 and Table 5 and Figure 1 and Figure 2).

All-cause mortality was higher at 20.7% in the intermediate to high probability of PH group compared to 5.4% in the low probability of PH group (*p* = 0.041). There was no difference in all-cause mortality between patients who were on immunosuppression vs. those who were not on immunosuppression in patients with intermediate to high probability of PH (33.3% vs. 7.1%; *p* = 0.169). However, all patients (irrespective of PH probability) on an immunosuppressant had 85.7% lower odds of death (B = −1.946; *p* = 0.021) compared to those not on immunosuppression. The intermediate to high probability of PH group also had a higher rate of lung transplantation at 6.9% compared to 0% in the low probability of PH group (*p* = 0.049) (Table 7).

## 4. Discussion

IIM and ILD are known to play a significant role in patients with MSA/MAA, but the prevalence of PH in patients with these antibodies remains unclear. PH has been associated with dysfunction of the immune system, and immunosuppression is an integral part of treatment for patients with MSA/MAA. To the best of our knowledge, this is the first study to assess the prevalence of the echocardiographic probability PH in patients with MSA/MAA and to examine the role of immunosuppression. Our main findings include the following:

1. Greater than a third of patients with positive MSA/MAA had intermediate to high probability of PH defined on TTE by TRV.
2. Patients with intermediate to high probability of PH had a higher prevalence of dermato-specific antibodies.
3. There was no difference in all-cause mortality between patients who were on immunosuppression and those who were not on immunosuppression in patients with intermediate to high probability of PH.

While data are limited on the prevalence of PH in patients with MSA/MAA, our study found that greater than a third of patients with positive MSA/MAA had intermediate to high probability of PH defined on TTE by TRV. The reported prevalence of PH in patients with PM or DM ranges from 6.2 to 63.6% using TTE evidence of PH [10]. There are case reports that describe the relationship seen with PH and PM/DM. The most common clinical manifestations are ILD, respiratory muscle weakness and infection. Patients described in this case series had progressive disease with PH that was poorly responsive to vasoactive agents [11]. Our study evaluated patients with a diagnosis of PM, DM, or ASSD. ASSD is the association of ILD and myositis with different anti-tRNA-synthetase antibodies. It is also characterized by Raynaud’s phenomenon, skin involvement, and arthritis. PH has been described in about 8% of patients with ASSD [12]. Given the scarcity of data on prevalence of PH in patients with MSA/MAA, our study is vital in elucidating this information further. In addition, our study also included additional antibodies such as MDA5, Mi2, TIF1, and NXP2 amongst others which not been included in prior studies examining prevalence of PH in this population [13]. Despite the high echocardiographic probability of PH in this population we understand that TRV is often inaccurate in identifying PH. For instance, in the study of ILD patients by Keir et al. [13], 40% of patients with low echocardiographic probability of PH (defined as TRV < 2.8 m/s) eventually had PH confirmed by RHC [12]. Nonetheless, TTE remains a useful tool as findings such as RV enlargement, RV systolic dysfunction, and TRV > 3.4 m/s increase the probability of PH by RHC [13]. Furthermore, these echocardiographic variables are independently associated with mortality in group 3 PH [13,14,15].

Although our study had limited RHC data, there was a significant difference noted in RAP/PAWP between the low and intermediate to high probability of PH group. This ratio has been found to be a specific predictor of mortality compared to other hemodynamic variables. Patients with higher RAP/PAWP have higher mortality, and this indicates RV failure. Our study further adds to the evidence that this ratio should be used in clinical prognostication and predictive models of PAH [16]. The cause of death in patients with PAH-SSc have been described as PAH, infection, and acute renal failure with up to a third of patients dying within 2 years of diagnosis [17]. In contrast, predictive factors for PAH leading to a higher probability of death in patients with myositis have been described as age, infection, IL-17A level, anti-SRP antibody, and steroid monotherapy [18]. Our group of patients with higher probability of PH also had higher rate of mortality. The demographic factors that were associated with higher probability of PH in our group of patients were male sex, smoking history, and ILD. Although ILD plays a significant role in patients with IIM, PAH can also exist without ILD. One case series described 3/9 (33.3%) of patients with IIM found to have a diagnosis of PAH without ILD, raising awareness of the diagnosis of PAH in IIM without ILD [19]. Our cohort of patients also had 2/29 (6.9%) of patients with higher probability of PH without a diagnosis of ILD.

Studies have also evaluated the prevalence of MSA/MAA in patients with PH. For example, Tobal et al. [3] evaluated 121 patients with PH and found a significantly higher prevalence of MSA/MAAs. Anti-synthetase and anti-overlap syndrome MAA were the most common [3]. Similarly, Sanges et al. [15] screened 5223 patients with group 1 PH, and included 34 patients with IIM, and excluded 31 who had extensive ILD, or an overlap syndrome reporting 3 patients with IIM and PAH. These patients had SSA antibodies more often than MSA [15]. Both studies contrast from our study, which found a higher prevalence of dermato-specific antibodies. These differences may have been from inclusion of ILD or locations (United States vs. France). Another potential reason is that our study used echocardiographic probability of PH while the study by Tobal et al. [3] used data derived by RHC. Nonetheless, our study adds to the growing body of evidence of PH in this population, further research is needed to investigate the role of the presence of dermato-specific antibodies as a predictor of PH in these patients.

Evaluation of the role of the immune system is imperative in determining the optimal treatment for patients with CTD-PAH. Immune cells such as T and B lymphocytes, macrophages, mast cells and dendritic cells are present around remodeled pulmonary arteries in the lungs of patients with PAH. This implies that immune cells may contribute to the development of PAH [20,21]. Th1 and Th17 cells may produce IL-6, IL-2, IL-21, interferon-gamma, and tumor necrosis factor alpha, which can cause the inflammatory and autoimmune response seen in PAH. Tregs suppress the inflammation seen in PAH, and a decreased Treg activity could increase the severity of PAH. Animal studies such as athymic mice or rats which lack T cells develop more severe PAH compared to normal rodents [22,23]. B cells play a crucial role in the pathogenesis of PAH by transforming into plasma cells which produce autoantibodies and through antigen presentation, cytokine production, differentiation of effector T cells, and collaboration with antigen presenting dendritic cells [24,25]. A cohort study of CTD-PAH patients showed improved pulmonary hemodynamics and survival time in patients receiving 12–18 months of high-dose cyclophosphamide and prednisone. However, there are no guidelines in place for recommended dosage of glucocorticoids for treatment of PAH due to lack of randomized clinical trials [26]. A single-center randomized control phase II clinical trial has shown the safety and efficacy of tacrolimus in CTD- PAH by showing improvement in 6MWD and echocardiographic parameters. [27]. These studies support the use of immunosuppression in patients diagnosed with CTD-PAH and show the importance of further investigation on the role of immunosuppression in patients with MSA/MAA and PAH. While studies support the use of immunosuppression in patients with CTD-PAH such as SLE [28,29], the role of immunosuppression for patients with MSA/MAA with PAH remains unclear. Our study found that there was no difference in all-cause mortality between patients who were on immunosuppression vs. those who were not on immunosuppression in patients with intermediate to high probability of PH. However, all patients (irrespective of PH probability) on an immunosuppressant had 85.7% lower odds of death compared to those not on immunosuppression showing an overall protective effect of immunosuppression in these patients. Probability of PH and immunosuppression had no overall effect on 6MWT distance walked and change in dyspnea score. However, there was a significant model effect of PH probability and immunosuppression on oxygen used. This significant finding shows that importance of further research into the role of immunosuppression as there could be a potential effect on the use of oxygen for these patients. Our study found that FVC and DLCO were lower in patients with intermediate to high probability of PH. Our patients also had higher all-cause mortality and higher rates of lung transplantation. The high morbidity and mortality seen with our patients who are commonly treated with immunosuppression must be investigated further to determine optimal treatment.

Our study has several limitations. It is limited by its retrospective design at a single tertiary care center with sicker patients. Therefore, our data may not be generalizable to the community. We also included echocardiographic data for probability of PH with patients who had a documented TRV instead of confirmed PH by RHC. Data from patients at a tertiary care center with sicker patients using echocardiographic data may have also led to overestimation of the prevalence of PH in our cohort of patients. However, there was a significant difference in TAPSE with the intermediate to high probability PH group having a lower TAPSE further supporting a diagnosis of PH by echocardiographic parameters. Also, TRV is a key indicator for PH and majority of patients with MSA/MAA do not undergo invasive testing with RHC. Using echocardiography with TRV as an estimate of PH is an important non-invasive way to diagnose and assess severity of PH. We also did not find differences between groups for 6MWT walked; oxygen used and change in dyspnea score. These outcomes are likely from the smaller size of our study. Our study also found a lower FEV1 in patients who were on immunosuppression, also likely from the small sample size of only 18 patients who had documentation of TRV on TTE, PFTs, and whether they were or were not on immunosuppression.

In summary, data are limited on the prevalence of PH and the role of immunosuppression in patients with MSA/MAA. In our group of patients, we found there is a higher prevalence for probability of PH defined by TRV on TTE in patients with MSA/MAA with reduced lung function, higher mortality, and greater rates of lung transplantation. Further research is needed to explore the role of dermato-specific antibodies with the development of PH. There may be an overall protective effect of immunosuppression but not specifically in patients with higher probability of PH. Further research with multi-cohort prospective studies is crucial in helping to guide therapy for these patients.

## Figures and Tables

**Table 1:** Patient demographics of whole sample and grouped differences between low probability of PH and intermediate to high probability of PH. Fisher’s Exact Test was used for statistical analysis for all variables except for age and BMI where T-test was used. Statistical significance is defined as *p*-value < 0.05.

		Total (*n* = 85)	Low (*n* = 56)	Intermediate-High (*n* = 29)	*p*-Value
Demographics					
Age (years)		56.5 ± 16.9	54.1 ± 17.0	61.2 ± 15.6	0.065
Sex *n* (%)	Male	28 (32.9%)	12 (42.8%)	16 (57.1%)	**0.002**
	Female	57 (67.1%)	44 (77.2%)	13 (22.8%)	
Race *n* (%)	White	49 (57.6%)	30 (61.2%)	19 (38.8%)	0.383
	AA	21 (24.7%)	13 (61.9%)	8 (38.1%)	
	Asian	4 (4.7%)	3 (75.0%)	1 (25.0%)	
	Hispanic	6 (7.1%)	5 (83.3%)	1 (16.7%)	
	Other	5 (5.9%)	5 (100.0%)	0 (0.0%)	
BMI (kg/m^2^)		29.5 ± 7.3	29.8 ± 6.9	28.9 ± 8.2	0.610
Smoking History (current or former) *n* (%)	Yes	27 (31.7%)	12 (21.4%)	15 (51.7%)	**0.004**
Diagnosis and Comorbidities					
Antisynthetase *n* (%) Dermatomyositis *n* (%) Polymyositis *n* (%) Undifferentiated *n* (%) OSA *n* (%)	Yes Yes Yes Yes Yes	23 (27.1%) 16 (18.8%) 7 (8.2%) 39 (45.9%) 35 (41.2%)	17 (30.4%) 9 (16.1%) 4 (7.1%) 26 (46.4%) 24 (42.6%)	6 (20.7%) 7 (24.1%) 3 (10.3%) 13 (44.8%) 11 (37.9%)	0.443 0.392 0.686 1.000 0.662
ILD *n* (%)	Yes	69 (81.2%)	42 (75.0%)	27 (93.1%)	**0.043**

**Table 2:** Patient descriptions of, outcomes, antibodies, therapies of whole sample and grouped differences between low probability of PH and intermediate to high probability of PH. Fisher’s Exact Test was used for statistical analysis. Statistical significance is defined as *p* value < 0.05.

	Total (*n* = 85)	Low (*n* = 56)	Intermediate-High (*n* = 29)	*p*-Value
Antibody				
MSA present *n* (%)	41 (48.2%)	23 (41.1%)	18 (62.1%)	0.073
MAA present *n* (%)	39 (45.9%)	28 (50.0%)	11 (37.9%)	0.361
Derm Ab present *n* (%)	18 (21.2%)	8 (14.2%)	10 (34.5%)	**0.048**
ASSD Ab present *n* (%)	27 (31.7%)	20 (35.7%)	7 (24.1%)	0.332
Jo1 *n* (%)	13 (15.3%)	9 (16.1%)	7 (24.1%)	0.392
PL7 *n* (%)	0 (0.0%)	0 (0.0%)	0 (0.0%)	1.000
MDA5 *n* (%)	11 (12.9%)	4 (7.1%)	7 (24.1%)	**0.040**
Mi2 *n* (%)	2 (2.4%)	1 (1.8%)	1 (3.4%)	0.569
TIF1 *n* (%)	2 (2.4%)	2 (3.6%)	0 (0.0%)	0.545
NXP2 *n* (%)	0 (0.0%)	0 (0.0%)	0 (0.0%)	1.000
PL12 *n* (%)	3 (3.5%)	3 (5.4%)	0 (0.0%)	0.548
EJ *n* (%)	2 (2.4%)	1 (1.8%)	0 (0.0%)	0.659
SAE *n* (%)	5 (5.9%)	1 (1.8%)	4 (13.8%)	**0.044**
SRP *n* (%)	6 (6.7%)	4 (7.1%)	2 (6.9%)	0.668
SSA 60 *n* (%)	6 (6.7%)	4 (7.1%)	2 (6.9%)	0.668
SSA 52 *n* (%)	23 (27.1%)	15 (26.8%)	8 (27.6%)	0.566
SSB *n* (%)	7 (8.2%)	5 (8.9%)	2 (6.8%)	0.552
KU *n* (%)	4 (4.7%)	4 (7.1%)	0 (0.0%)	**0.047**
OJ *n* (%)	5 (5.9%)	4 (7.1%)	1 (3.4%)	0.255
U1RNP *n* (%)	3 (3.5%)	3 (5.4%)	0 (0.0%)	0.548
PMScl *n* (%)	3 (3.5%)	0 (0.0%)	3 (10.3%)	**0.037**
Th/To *n* (%)	1 (1.2%)	0 (0.0%)	1 (3.4%)	0.341

**Table 3:** TTE and RHC of whole sample and grouped differences between low probability of PH and intermediate to high probability of PH. T-test was used for statistical analysis. Statistical significance is defined as *p* value < 0.05.

	Total	Low	Intermediate–High	*p*-Value
TTE				
LA Size (cm)	3.8 ± 0.7	3.8 ± 0.7	3.9 ± 0.8	0.581
*n*	71	48	23	
RVSP (mmHg)	35.5 ± 7.0	33.6 ± 3.8	44.4 ± 11.6	0.105
*n*	28	23	5	
TAPSE (mm)	21.4 ± 5.9	23.0 ± 5.2	18.1 ± 6.2	**0.029**
*n*	30	20	10	
RHC				
RAP (mmHg)	8.5 ± 4.3	7.6 ± 3.5	9.0 ± 6.6	0.502
*n*	20	8	12	
RV Systolic (mmHg)	46.8 ± 21.1	45.5 ± 20.3	47.7 ± 22.4	0.829
*n*	20	8	12	
RV Diastolic (mmHg)	8.8 ± 5.8	9.0 ± 4.9	8.6 ± 5.4	0.879
*n*	20	8	12	
PAOP (mmHg)	11.5 ± 3.4	11.1 ± 3.7	11.7 ± 3.3	0.736
*n*	20	8	12	
TDCO (L/min)	4.6 ± 1.0	4.6 ± 1.1	4.6 ± 1.0	0.985
*n*	20	8	12	
MPAP (mmHg)	28.0 ± 13.0	27.3 ± 13.1	28.5 ± 13.5	0.840
*n*	20	8	12	
CI (L/min/m^2^)	2.5 ± 0.7	2.5 ± 0.6	2.4 ± 0.7	0.803
*n*	20	8	12	
PVR (WU)	5.1 ± 3.3	3.9 ± 2.8	6.5 ± 3.8	0.273
*n*	9	5	4	
Stroke Volume (mL)	65.8 ± 21.3	61.5 ± 25.3	69.0 ± 18.6	0.470
*n*	19	8	11	
RAP/PAWP	0.8 ± 0.4	0.6 ± 0.2	0.9 ± 0.4	**0.048**
*n*	18	8	10	

**Table 4:** 6MWT and Pulmonary Function Test of whole sample and grouped differences between low probability of PH and intermediate to high probability of PH. T-test was used for all variables except oxygen use where Fisher’s Exact Test was used. Statistical significance is defined as *p* value < 0.05.

	Total	Low	Intermediate–High	*p*-Value
6 min Walk Distance (m)	343.0 ± 128.0	363.2 ± 115.6	294.9 ± 147.5	0.108
*n*	44	31	13	
Oxygen (Yes)	37 (86.0%)	26 (86.7%)	11 (84.6%)	0.599
*n*	43	30	13	
Dyspnea Score (Change)	2.7 ± 2.1	2.8 ± 2.2	2.5 ± 1.8	0.629
*n*	43	30	13	
FEV1 (L)	69.9 ± 19.8	72.9 ± 19.9	62.5 ± 17.7	0.058
*n*	64	46	18	
FVC (L)	67.8 ± 21.6	71.3 ± 22.4	58.8 ± 16.7	**0.037**
*n*	65	47	18	
DLCO (mL/min/mmHg)	51.6 ± 21.9	56.3 ± 21.8	36.9 ± 15.5	**0.003**
*n*	58	44	14	
TLC (L)	66.3 ± 20.3	69.6 ± 20.7	55.6 ± 15.1	0.057
*n*	43	33	10	

**Table 5:** 6MWT and Pulmonary Function Test of those with intermediate–high probability of PH and grouped differences between those on immunosuppression vs. those who are not. *t*-test was used for all variables except oxygen use where Fisher’s Exact Test was used. Statistical significance is defined as *p* value < 0.05.

	Total (Intermediate–High)	No Immunosuppressant	Immunosuppressant	*p*-Value
6 min Walk Distance (m)	294.9 ± 147.5	289.2 ± 142.2	298.5 ± 160.3	0.918
*n*	13	5	8	
Oxygen (Yes)	11 (84.6%)	4 (80.0%)	7 (87.5%)	0.999
*n*	13	5	8	
Dyspnea Score (Change)	2.5 ± 1.8	2.2 ± 1.6	2.6 ± 1.9	0.691
*n*	13	5	8	
FEV1 (L)	62.5 ± 17.7	72.1 ± 5.5	56.3 ± 20.2	**0.030**
*n*	18	7	11	
FVC (L)	58.8 ± 16.7	64.7 ± 9.2	55.0 ± 19.5	0.240
*n*	18	7	11	
DLCO (mL/min/mmHg)	36.9 ± 15.5	34.3 ± 3.5	38.3 ± 19.4	0.667
*n*	14	5	9	
TLC (L)	55.6 ± 15.1	56.0 ± 12.2	55.4 ± 18.0	0.958
*N*	10	4	6	

**Table 6:** Effect of immunosuppression and Echo Probability of PH on 6MWT parameters. ANOVA was used for statistical analysis. Statistical significance is defined as *p* value < 0.05.

Effect	Sum of Squares	df	F-Statistic	*p*-Value
6MWT				
a. Probability Group	40,733.1	1	2.5	0.12
b. Immunosuppression	573.7	1	0.1	0.851
Model (a x b)	43,222.5	2	1.3	0.273
Dyspnea Score				
a. Probability Group	1.1	1	0.3	0.619
b. Immunosuppression	0.2	1	0.1	0.84
Model (a x b)	1.2	2	0.1	0.873
Oxygen Use				
a. Probability Group	13.6	1	3.9	0.053
b. Immunosuppression	6.5	1	1.9	0.174
Model (a x b)	22.9	2	3.3	**0.045**

**Table 7:** Patient descriptions of comorbidities, outcomes, antibodies, therapies of whole sample and grouped differences between low probability of PH and intermediate to high probability of PH. Fisher’s Exact Test was used for statistical analysis. Statistical significance is defined as *p* value < 0.05.

	Total (*n*= 85)	Low Probability (*n* = 56)	Intermediate-High Probability (*n* = 29)	*p*-Value
Outcome				
All-cause Mortality *n* (%)	9 (10.6%)	3 (5.4%)	6 (20.7%)	**0.041**
Lung Transplantation *n* (%)	2 (2.4%)	0 (0.0%)	2 (6.9%)	**0.049**

## Data Availability

The original contributions presented in this study are included in the article. Further inquiries can be directed to the corresponding author.
